# The neurobiological mechanisms and therapeutic prospect of extracellular ATP in depression

**DOI:** 10.1111/cns.14536

**Published:** 2024-02-20

**Authors:** Kaixin Wang, Shiqian Huang, Daan Fu, Xinxin Yang, Lulin Ma, Tianhao Zhang, Wenjing Zhao, Daling Deng, Yuanyuan Ding, Yanyan Zhang, Li Huang, Xiangdong Chen

**Affiliations:** ^1^ Department of Anesthesiology, Union Hospital, Tongji Medical College Huazhong University of Science and Technology Wuhan China; ^2^ Institute of Anesthesia and Critical Care Medicine, Union Hospital, Tongji Medical College Huazhong University of Science and Technology Wuhan China; ^3^ Key Laboratory of Anesthesiology and Resuscitation (Huazhong University of Science and Technology), Ministry of Education Wuhan China

**Keywords:** anti‐depressant therapy, astrocytes, depression, eATP, MDD, mitochondria, P2X7R, purinergic receptors

## Abstract

**Background:**

Depression is a prevalent psychiatric disorder with high long‐term morbidities, recurrences, and mortalities. Despite extensive research efforts spanning decades, the cellular and molecular mechanisms of depression remain largely unknown. What's more, about one third of patients do not have effective anti‐depressant therapies, so there is an urgent need to uncover more mechanisms to guide the development of novel therapeutic strategies. Adenosine triphosphate (ATP) plays an important role in maintaining ion gradients essential for neuronal activities, as well as in the transport and release of neurotransmitters. Additionally, ATP could also participate in signaling pathways following the activation of postsynaptic receptors. By searching the website PubMed for articles about “ATP and depression” especially focusing on the role of extracellular ATP (eATP) in depression in the last 5 years, we found that numerous studies have implied that the insufficient ATP release from astrocytes could lead to depression and exogenous supply of eATP or endogenously stimulating the release of ATP from astrocytes could alleviate depression, highlighting the potential therapeutic role of eATP in alleviating depression.

**Aim:**

Currently, there are few reviews discussing the relationship between eATP and depression. Therefore, the aim of our review is to conclude the role of eATP in depression, especially focusing on the evidence and mechanisms of eATP in alleviating depression.

**Conclusion:**

We will provide insights into the prospects of leveraging eATP as a novel avenue for the treatment of depression.

## Graphical abstract

The knock out of adenosine triphosphate (ATP) release channel (Panx1, Calhm2) and IP3R2, which could lead to the decreased extracellular ATP concentration, will exert depressive‐like effects. This means extracellular ATP may play an important role in depression. Purinergic receptors play vital roles in depression. The activation of P2X2R and P2X4R may play important roles in anti‐depressant effects, while P2X7R signaling could potentially promote the development of depressive‐like behaviors. Both A1 receptors and A2a receptors have been found to play a significant role in depression. A2a receptor antagonists have been reported to have clear anti‐depressant effects.

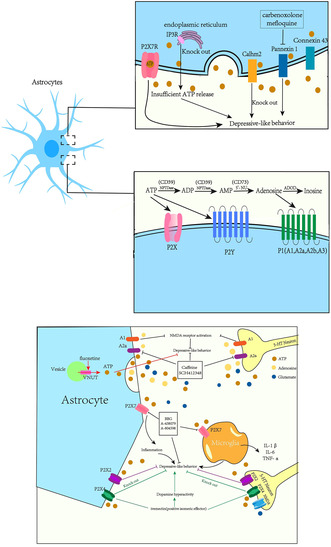

## 1 INTRODUCTION

Depression is a complex, prevalent, and severe mental disease^1^ that exerts a detrimental impact on daily activities and diminishes the quality of life for millions of people worldwide. By 2030, it is projected to rank among the top three contributors to the disease burden,^2^ and a significant cause of disability and suicidal behavior.^3^ Depression, characterized by low mood, feelings of hopelessness, loss of happiness, and lack of motivation, affects up to 350 million people worldwide.^4^, ^5^ The probability of women suffering from depression is more than 30% while that of men is more than 15%, thereby imposing substantial social and economic burdens.^6^ Sadly, about one third of individuals with depression do not have effective anti‐depressant treatment.^7^, ^8^

The pathophysiologic cause of depression is unknown, and currently, there are no clinically useful biological diagnostic markers or biological screening tests available.^9^ However, several recognized risk factors should be taken into consideration, such as low socioeconomic status; comorbid chronic medical conditions, such as diabetes, cardiovascular disease, or obesity; and a personal or family history of major depressive disorder (MDD).^9^ MDD, is a highly prevalent subtype of clinical depression, and accounts for 4.4% of the global disease burden.^10^

Initial treatment for MDD may involve anti‐depressant medication, psychotherapy, or a combination of both. Complementary, alternative, and exercise treatments may also be used but have a more limited evidence base.^11^, ^12^ Typically, treatment begins with a second‐generation anti‐depressant (selective serotonin reuptake inhibitor [SSRI]; serotonin–norepinephrine reuptake inhibitor; or atypical anti‐depressant like mirtazapine or bupropion).^9^ However, it is worth noting that the crucial Sequenced Treatment Alternatives to Relieve Depression (STAR*D) study revealed that one third of MDD patients had not achieved remission after four consecutive anti‐depressant trials.^13^ Pharmacological treatment resistance to anti‐depressant therapy is one of the most challenging situations in the clinical management of affective disorders. According to the recent widely accepted definition, treatment‐resistant depression (TRD) is characterized by an inadequate response to at least two trials of anti‐depressant treatment at adequate dose and duration when administered as monotherapy.^14^, ^15^

Therapeutic strategies to improve inadequate anti‐depressant response in TRD patients usually begin with the exclusion of pseudo‐resistance; Augmentation with second‐generation antipsychotics and lithium seem to serve as the treatment options supported by the strongest augmentative evidence in TRD. Nevertheless, the augmentative potential of using anticonvulsive drugs, nutraceuticals, and glutamatergic as well as anti‐inflammatory agents remains incompletely verified and needs further research. About other somatic therapies, stimulation therapies, particularly electroconvulsive therapy and repetitive transcranial magnetic stimulation, have been found to be effective in TRD.^16^, ^17^ So far, intranasal S‐ketamine treatment, the sole treatment option specifically indicated in TRD,^18^ is an adjunctive therapy to anti‐depressants targeting the monoaminergic system. Additionally, many novel investigational drugs and regimens are undergoing clinical trials (see in the review^19^).

Brain regions associated with depression include the medial prefrontal cortex (mPFC), the amygdala, hippocampus,^20^ and the lateral septum.^21^, ^22^, ^23^ An important pathophysiological feature of depression is the disturbance of energy metabolism.^24^, ^25^, ^26^ Positron emission tomography examination has shown the reduced blood flow and impaired glucose metabolism in caudate nucleus, anterior cingulate cortex, and prefrontal cortex of depressed patients.^24^, ^26^ In depressive mouse models, characteristics of abnormal energy metabolism have also been documented, including abnormal glucose metabolism, elevated lactate level, mitochondrial dysfunction,^27^, ^28^, ^29^, ^30^ and abnormal ATP level.^31^, ^32^, ^33^, ^34^

ATP is the biological energy currency and the primary driver of enzyme activity in all cells and tissues.^35^ Microglia could release large amounts of ATP, especially during neuroinflammation.^20^ It is broadly recognized that ATP not only supports energy storage within cells but also serves as a rapidly excitatory neurotransmitter or neuromodulator that mediates purinergic signaling in cell proliferation, differentiation, and death, both in healthy individuals and patients with brain disease.^36^, ^37^ ATP can be released from astrocytes, microglia, neurons, endothelial cells, and smooth muscle cells (present in vessels). Intracellularly, ATP concentration ranges around 5 mM, while in the interstitial space of normal nonstressed tissues, its concentration is in the nanomolar rang.^38^, ^39^ Under normal circumstances, extracellular ATP (eATP) concentrations are much smaller than intracellular levels, and a state of equilibrium is maintained. In some diseases, eATP is significantly elevated, with concentrations exceeding 100 μ mol/L, far above normal eATP levels. ATP released outside the cell is short‐lived and is usually rapidly degraded by ecto‐nucleosidase to adenosine diphosphate (ADP), adenosine monophosphate (AMP), and adenosine.

eATP is an important medium of astrocyte‐neuron communication and has been linked to depressive‐like disorders in rodent models.^40^, ^41^ Substantial evidence from animals suggests that insufficient ATP release from astrocytes may contribute to the development of MDD.^42^, ^43^, ^44^ Clinical studies on depression also indicate a potential reduction in cellular ATP levels, which could also be observed in rodents with corticotropin‐releasing hormones and stress‐induced depressive‐like behavior.^45^ Furthermore, studies have shown supplementing ATP can alleviate stress‐induced depressive‐like behaviors in mice, especially in those with Apolipoprotein E4 (APOE4) targeted replacement.^46^ It is worth noting that anti‐depressants have been reported to increase eATP levels.^47^ In this review, we focus on summarizing the anti‐depressant effects of eATP. and also list controversial views aiming to illustrate the critical role of eATP in the occurrence and development of depression.

**FIGURE 1:**
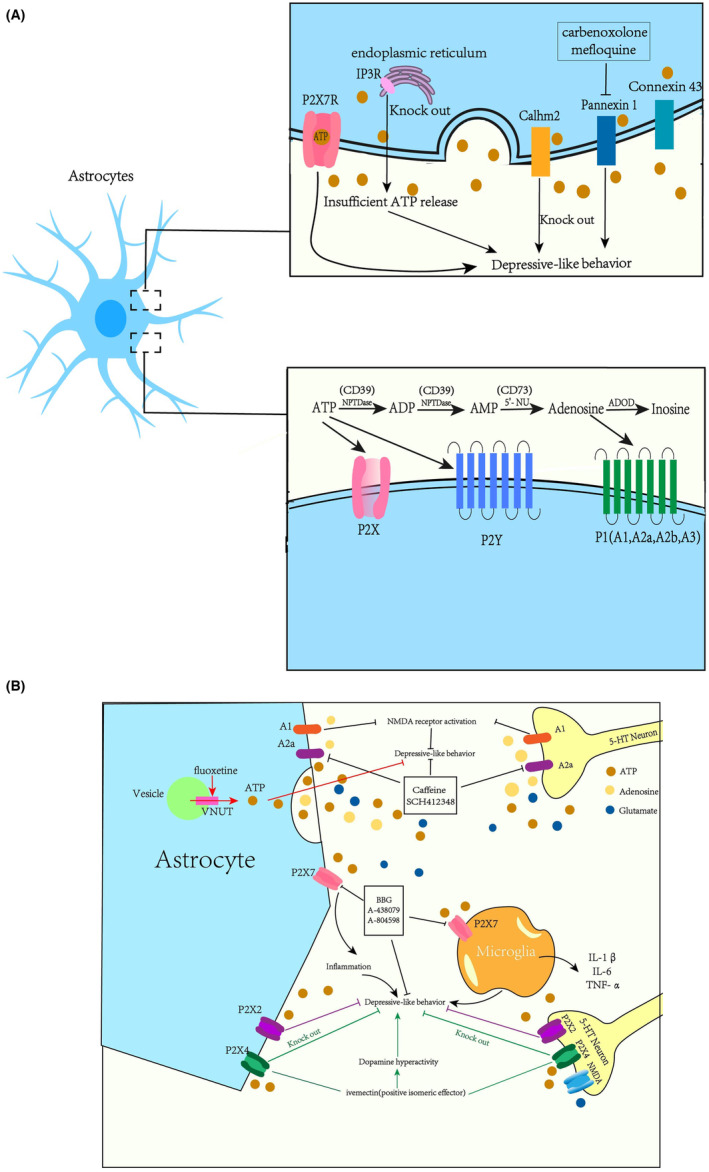
(A) This picture concludes the relationship between extracellular ATP (eATP)and depression. (B) This picture concludes the relationship between eATP and depression. The activation of ATP release channel (Panx1,^50^ Calhm2^52^) which could lead to the increased eATP concentration will exert anti‐depressant effects. Anti‐depressant fluoxetine acted on astrocytes and mediated its therapeutic effects by facilitating vesicular nucleotide transporter (VNUT)‐dependent ATP exocytosis.^56^ ATP release was elevated in calorie‐restricted mice but decreased in IP3R2‐KO mice in depression‐related brain regions.^43^ P2X2R^57^, ^58^ and P2X4R^59^, ^60^ may play an important role in anti‐depressant effects. Stress may trigger excessive ATP release, which in turn activates P2X7R signaling, potentially promoting the development of depressive‐like behaviors which can be blocked by P2X7R antagonists (e.g., BBG,^54^, ^55^, ^61^ A‐438079,^61^, ^62^ A‐804598^53^). Both A1 receptors and A2a receptors have been found to play an important role in depression. Adenosine antagonists, such as caffeine, the non‐selective adenosine antagonist, or SCH412348, the selectively A2a receptor antagonist have clear anti‐depressant effect in behavioral paradigms in the FST and TST.^63^, ^64^, ^65^ “↑” promote, “˩” inhibit. Abbreviations: CR, caloric restriction; ATP, adenosine triphosphate; ADP, adenosine diphosphate; AMP, adenosine monophosphate; CD39, Ectonucleoside triphosphate diphosphohydrolase‐1; CD73, Ecto‐5’‐Nucleotidase; iL ‐1, interleukin‐1; iL‐6, interleukin‐6; TNF‐α, tumor necrosis factor‐α; Calhm2, calcium homeostasis modulator 2; Panx1, *Pannexin1*; IP3R, inositol 1,4,5‐trisphosphate receptors; VNUT, Vesicular nucleotide transporter; P2Y11, a kind of purinergic receptors; SCH412348, a kind of selective A2A receptor antagonist; BBG, brilliant blue G; A‐438079, a kind of P2X7R antagonist; A‐804598, a kind of P2X7R antagonists; FST, forced swimming test; TST, tail suspension tests.

We have made a Table 1 and Figure 1 to visually present our findings.

**TABLE 1:** Studies about the relationship between eATP and depression in various animal models.

References	Models	Animals	Results	Conclusions
[46]	CUMS	C57BL/6J	Apolipoprotein E4 (APOE ε4) allele mediates depressive‐like behaviors in mice in an age‐dependent manner	In clinical settings, ATP supplementation may be effective for elderly depression patients with APOE4 carrier
[48]	CSDS	C57BL/6J mice	Hippocampal extracellular ATP (eATP) concentration↓, hippocampal neurogenesis, and dendritic spine numbers in defeated mice↓	Hippocampal CD39 contributes to CSDS‐induced depressive‐like behavior via hydrolyzing eATP, indicating that CD39 may be a promising new target for the treatment of depression
[49]	Restraint stress	Male Wistar rats	Increasedsynaptic ATP release coupled to CD73‐mediated formation of extracellular adenosine contributes to mood and memory dysfunction triggered by repeated restraint stress	This prompt considers interventions decreasing ATP release and CD73 activity as novel strategies to mitigate the burden of repeated stress
[50]	CSDS	Male C57BL/6J mice	The expression and function of Panx1 in the medial prefrontal cortex (mPFC) of susceptible mice↓	The downregulation of Panx1 in CSDS model results in lacking of eATP in the mPFC and then contributes to depressive‐like behaviors
[51]	CSDS	Male C57BL/6J mice	CSDS Deletion of Ephx2 in adult astrocytes induces resilience to stress	EET signaling in astrocytes in the mPFC is essential for behavioral adaptation in response to psychiatric stress
[43]	CR	Male C57BL/6 mice	Deletion of IP3R2 blocked the anti‐depressant‐like effect induced by calorie restriction	Astrocyte ATP in the mPFC plays a role in the anti‐depressant‐like effect induced by CR, possibly by decreasing the excitability of the mPFC
[52]	Calhm2‐KO	Male C57BL/6 mice	Calhm2 KO models	Calhm2 acts as a critical ATP‐releasing channel that modulates neural activity and as a potential risk factor of depression
			ATP concentrations ↓	
			Hippocampal spine number ↓, neural dysfunction	
[53]	CUS	Male/n.a.	CUS resulted in(a) reduced consumption in the SPT, elevated latency to feed in the NSFT, and reduced time in the open arms of the EPM. (b) A‐804598 significantly reversed the CUS effects in the SPT, NSFT, and EPM	Psychological “stress” is sensed by the innate immune system in the brain via the ATP/P2X7R–NLRP3 inflammasome cascade, and they identify novel therapeutic targets for the treatment of stress‐related mood disorders and comorbid illnesses
		Rat/Sprague–Dawley		
[54]	Unpredictable Chronic Mild Stress (UCMS)	Male	UCMS resulted in (a) impaired CSS and NBS at weeks 8 and 9; improved by BBG. (b) increased microglial activation and P2X7R expression in limbic and cortical areas; ameliorated by BBG. (c) HPA dysfunction; attenuated by BBG	P2X7R is involved in recovery from depressive‐like states caused by exposure to UCMS in a mechanism that involves restoration of the HPA axis but not hippocampal neurogenesis.
		Mouse/BALB/cByJ		P2X7R antagonist agents may have potential value in the pharmacological management of depression
[55]	UCMS	Male	UCMS resulted in: (a) anhedonic behavior in SPT; attenuated by 25 and 50 mg BBG. (b) elevated immobility in the FST; recovered by 50 mg but not 25 mg BBG. (c) mRNA level increase of P2X7R, caspase‐1, ASC, NF−ƙB, IL‐1β, IL‐6; attenuated by 50 mg BBG; microglia activation (Iba‐1 in IHC; hippocampus) in the UCMS group; reduced by both 25 and 50 mg BBG	Chronic administration of BBG in CUMS model results in anti‐depressant‐like activity in a dose‐dependent manner
		Rat/Wistar‐Albino		

*Note*: “↑” increase and “↓” decrease.

## 2 NEUROLOGICAL MECHANISM OF eATP IN DEPRESSION

### 2.1 Insufficient ATP release from astrocytes may lead to the occurrence of depression

Insufficient ATP release from astrocytes is believed to be closely associated with the onset of depression. Research has demonstrated that the loss of insulin (IR) signaling leads to a reduction in ATP exocytosecretion by astrocytes, contributing to the development of depression. Notably, giving ATP analogs can reverse depressive‐like behaviors in IR knockout (KO) mice.^66^ Postmortem histopathological studies consistently revealed a decrease in the density and number of glial cells in the mPFC of MDD patients.^18^, ^67^ Furthermore, a microarray‐based study has identified dysregulation of genes involved in ATP biosynthesis and utilization in the mPFC of MDD patients.^68^ Stimulating ATP release from astrocytes or infusing ATP into the mPFC has been shown to produce anti‐depressant‐like effects. However, the specific mechanism underlying inadequate ATP release from astrocytes in depression remains unclear. Some researchers have made efforts to unravel the intrinsic mechanism of ATP reversing depression, focusing on neural circuit and molecular pathways. Gao and colleagues reported that the ATP level in the mPFC regulates depressive‐like behaviors through the mPFC‐lateral habenula pathway (LHb),^57^ suggesting a potential neural circuit mechanism. Cao and colleagues reported that optogenetic activation of mPFC neurons or the mPFC‐striatum pathway can rescue disrupted resting‐state functional magnetic resonance imaging functional connectivity and depressive‐like behaviors in 1,4,5‐trisphosphate‐type‐2 receptor knockout mice (ITPR2−/− mice) with astrocytic calcium deficiency.^69^ Zhang et al. reported that genetic and pharmacological inhibition of astrocytic mysm1 could alleviate depressive‐like disorders by promoting ATP production and ATP release through the p53‐AMPK‐Sirt1‐PGC1α (p53, a kind of tumor suppressor gene; AMPK, AMP activates protein kinase; Sirt1, silent mating type information regulation 2 homolog‐1; PGC1α, peroxisome proliferators‐activated receptor γ coactivator l α) pathway.^70^ Lu et al. discovered that the reduction in astrocytic glucocorticoid receptors leads to decreased ATP release, resulting in depressive‐like behaviors, and this effect is mediated by the PI3K‐AKT (PI3K, Phosphatidylinositol3‐kinase; AKT/PKB, protein kinase B) signaling pathway.^71^ In summary, while it is clear that insufficient ATP release from astrocytes is linked to depression and that ATP supplementation can alleviate depressive symptoms, the specific mechanisms involved remain poorly understood. Ongoing research suggests that neural circuits and molecular pathways, such as the mPFC‐LHb circuit, p53‐AMPK‐Sirt1‐PGC1α, and PI3K‐AKT pathways, play crucial roles in modulating ATP release from astrocytes, shedding light on potential targets for further investigation.

Astrocytes are the most abundant type of glial cells in the brain^32^ and play a pivotal role in the pathophysiology of depression.^66^ eATP serves as a crucial mediator of astrocyte‐neuron communication,^72^, ^73^ which has been proven to be associated with depressive‐like disorders in rodents.^40^, ^41^ Numerous evidence have shown that ATP released from astrocytes influences neuronal activity through the astrocyte‐neuron tripartite synapse, dynamically regulating synaptic transmission of adjacent neurons.^74^, ^75^, ^76^ Astrocytes have been reported to secret ATP through several major pathways: (1) Connexin 30/43 can form gap junctions between astrocytes, while Pannexin 1 can assemble into half‐channels but could not form gap junctions between astrocytes, through which various signaling molecules such as glutamate, adenosine, Ca^2+^, and ATP can exchange with each other.^77^, ^78^, ^79^, ^80^, ^81^ (2) Astrocytes release ATP mainly through the max‐anion channel under hypoxic‐glucose deprivation.^82^ (3) Astrocytes also release ATP through the SNARE‐VAMP (SNARE, soluble N‐ethylmaleimide‐sensitive factor attachment protein receptor; VAMP, vesicle‐associated membrane protein) lysosomal pathway via Ca^2+^‐dependent exocytosis.^83^, ^84^, ^85^, ^86^ In the following sections, we will provide a detailed explanation of the ATP release mechanisms employed by astrocytes and establish the role of eATP in the onset of depression from this perspective.

#### 2.1.1 ATP release channels (Calhm2/Panx‐1)

The membrane protein Calhm2 (calcium homeostasis modulator 2), functioning as an anion channel, controls the release of ATP from astrocytes, without dependence on lysosomes and gap junctions.^52^ Studies have shown that intraperitoneal injection of ATP significantly elevates ATP levels in the hippocampus of Calhm2‐KO mice and ameliorates the depressive‐like behavior observed in these mice. The duration of struggling in the forced swimming test and tail suspension test is prolonged, possibly due to the restoration of the reduced number of dendritic spines.^52^ Pannexin‐1(Panx‐1), a plasma membrane channel that allows molecules like ATP to pass through, can have its depressive‐like effects blocked through eATP preconditioning when obstructed by gap junction blockers carbenoxolone and mefloquine in the mPFC region.^50^ This suggests a potential anti‐depressant role for eATP. However, contrary results have been reported, dye absorption experiments in hippocampal slices showed that acute stress (known to trigger depressive‐like behavior) could induce the opening of Connexin 43and Panx‐1 channels, leading to ATP release.^87^ This appears to contradict the previously cited evidence suggesting the anti‐depressant effects of ATP. An explanation for these contradictory results may be rooted in the fact that these conclusions are based on a review of different tissue structures and different regions of brain. In vitro research cannot completely simulate the physiological environment in vivo. What's more, different depressive animal models have different pathological bases, which may account for these contradictory findings.

#### 2.1.2 Itpr2‐/IP3R2‐Ca^2+^‐ATP pathway

Astrocytes are thought to regulate neuronal excitability by releasing ATP in a calcium‐dependent manner.^88^ The inositol 1,4,5‐trisphosphate receptors (IP3Rs) located on the endoplasmic reticulum of cells are key mediators in regulating the release of calcium ions.^43^ The protein encoded by the ITPR2 gene belongs to the IP3R family. Research has demonstrated that both ITPR2^‐^deletion and blocking vesicular neurotransmitter transmission could lead to inadequate ATP release from astrocytes, resulting in depressive‐like behaviors which can be alleviated by supplying ATP and stimulating astrocytes to release endogenous ATP.^41^ Additionally, this study reported that it is ATP rather than its hydrolytic product adenosine could reverse depressive‐like behaviors in ITPR2^−^ mice.^41^ Similarly, another paper suggested that the loss of IP3R2 blocked increased ATP levels in the mPFC of mice exposed to caloric restriction (CR), while this effect was absent in IP3R2‐KO mice,^43^ implying that the astrocyte IP3R2‐ATP pathway may play an important role in CR‐induced anti‐depressant effects.

#### 2.1.3 Vesicular nucleotide transporter

Nucleotide transport within cells cannot cross the cell membrane freely.^89^ Instead, it relies on vesicular storage and transportation.^90^ Some studies suggest that the treatment with SSRI can promote ATP release, resulting in the activation of the ATP receptors via VNUT and the increased brain‐derived neurotrophic factor (BDNF) expression in astrocytes.^56^ Both BDNF^40^ and SSRI^56^ have been confirmed to be closely related to depression. Furthermore, it is widely acknowledged that Lysosomes in astrocytes contain abundant ATP,^85^ and previous studies have shown the crucial role of astrocytic ATP release in depression.^41^ Soluble epoxide hydrolase, a key enzyme in epoxyeicosatrienoic acid (EET)signal transduction in the mPFC of susceptible mice, is mainly expressed on lysosomes of astrocytes and exhibits an anti‐depressant role.^42^ It is speculated that this may be due to the increased ATP release from astrocytes.

Briefly, there is ample evidence supporting the notion that ATP release from astrocytes could alleviate depression, with only a few studies putting forward the opposite view. The differences may depend on the specific situation. Whatever, these pieces of evidence collectively suggest that ATP release from astrocytes plays an important role in the onset and development of depression. Stimulating the release or replenishment of endogenous ATP in astrocytes may have an anti‐depressant effect. Furthermore, studies focusing on how stress reduces ATP release in astrocytes will enhance the comprehension of the pathophysiology of MDD.

### 2.2 Synaptically‐derived ATP and depression

ATP is released through various pathways, which include exocytosis from presynaptic terminals and diffusion through large transmembrane pores (e.g., hemichannels, P2X [7] receptors, or volume‐sensitive chloride channels) expressed in astroglia membranes.^91^ In presynaptic terminals, ATP is accumulated and stored in the synaptic vesicles.^91^

ATP derived from both glia and neuronal sources can pre‐modulate the efficacy of excitatory synapses and thereby can have an important role in the glia–neuron communications and brain meta‐plasticity.^92^ Synaptic plasticity disturbances are crucial in the onset and progression of depression, especially in the hippocampus, which is highly susceptible to stress and the most frequently studied in the context of depression.^93^ It is widely acknowledged that depression is considered as a Glial‐Based Synaptic Dysfunction,^40^ and synaptically‐derived ATP, which has the potential to restore synaptic plasticity, may play an important role in depression.

### 2.3 ATP released from neurons and depression

ATP serves not only as the “energy currency” of neurons, but also as a signaling molecule that facilitates information transfer between neurons and glial cells. ATP functions as a glial transmitter that mediates the neuron‐glial cell network interaction.^94^ eATP can be released from neurons both vesicularly^95^, ^96^ and non‐vesicularly.^97^ ATP could exhibit modulatory effects on neuronal functions, with mounting evidence suggesting its pivotal role in synaptic plasticity.^98^ Synaptic plasticity damages play a crucial role in the onset and development of depression, especially in the hippocampus, which is more susceptible to stress and the most frequently studied brain region in depression.^93^

The effects of extracellular synaptic ATP on synaptic plasticity are a composite of effects of P2 and P1(adenosine) receptors. Postsynaptic responses to ATP are mediated by metabotropic P2Y and ionotropic P2X receptors, which are abundantly expressed in neural cells. Strong impacts of P2X receptors on synaptic plasticity arise from their high calcium permeability, capability to interact with other receptors, and widespread exocytotic release of ATP from central neurons and glia.^99^ Generally, P2X‐mediated synaptic currents are not very large, rarely exceeding 50–100 pA in amplitude and accounting for 5%–15% of the synaptic currents mediated by glutamate. Nonetheless, the ATP‐mediated synaptic transmission can still be functionally significant. This is especially noteworthy because the activation of postsynaptic P2X receptors can facilitate Ca^2+^ entry at resting membrane potentials.^99^ The Application of ATP triggers substantial Ca^2+^ signals in central neurons, which are mediated by Ca^2+^ entry through P2X receptors/voltage‐gated Ca^2+^ channels and by P2Y‐mediated Ca^2+^ release from intracellular store.^100^, ^101^ It is widely reported that cytoplasmic Ca^2+^ signals are important for the synaptic potentiation and depression.^102^, ^103^, ^104^

Adenosine neuromodulation depends on a balanced activation of inhibitory A1(A1R) and facilitatory A2a receptors (A2aR). Both A1R and A2aR could modulate hippocampal glutamate release and NMDA‐dependent long‐term potentiation (LTP).^105^ While A2a receptors are predominantly found in the striatum, they are also present in the limbic system and neocortex.^106^, ^107^ Their presence in synapses suggests a role for A2a receptors in controlling synaptic transmission, either by presynaptic mechanisms or by regulating NMDA receptor function.^107^

It is speculated that neuron‐derived eATP could potentially play an anti‐depressant role by modulating synaptic plasticity.

### 2.4 ATP+P2XR

Purinergic signaling pathways play a crucial role in various psychiatric disorders, such as MDD.^108^, ^109^, ^110^, ^111^ These pathways are mediated by the action of nucleoside and nucleotide on P1 (P1R) and P2(P2R) receptors. P1R is a group of 4 different receptors (A1, A2A, A2B. and A3 receptors) and these receptors are all members of the G‐protein‐coupled family of 7‐transmembrane spanning receptors,^112^, ^113^ while P2R can be categorized into metabolic (P2Y receptor, P2YR) and ionotropic(P2X receptor, P2XR) subtypes, all of which are sensitive to ATP, ADP, uracil triphosphate and uracil diphosphate.^99^, ^114^, ^115^ Among purinergic receptors, P2X7R is considered a putative target for therapeutic intervention in mood disorders.^116^ A1 and A2a receptors are also considered potential targets for therapeutic intervention in mood disorders.^63^, ^64^, ^117^, ^118^

#### 2.4.1 ATP+P2X7R and depression

P2X7R, an ATP‐gated cation channel,^119^ is only activated by a high concentration of eATP (concentration for 50% of maximal effect (EC50) ≥ 100 μmol L^−1^) after exposure to stress.^120^ When activated, P2X7R allows Ca^2+^ and Na^+^ to flow inward and K^+^ to flow out of the cell.^121^ Stress may trigger excessive ATP release, which in turn activates P2X7R signaling in the hippocampus, potentially promoting the development of depressive‐like behaviors.^121^ Several studies have suggested that P2X7R is associated with depression, with behavioral findings of increased activity and decreased inactivity in P2X7R‐KO mice during forced swimming tests (FST) and tail suspension tests (TST).^122^, ^123^ This was further supported by another study showing the reduced depressive‐like behavior in P2X7R‐KO mice in the FST and the reduced use of anti‐depressants.^122^ Numerous studies have confirmed the anti‐depressant effects of P2X7R antagonists, such as blue brilliant G,^54^, ^55^, ^61^ A‐438079^61^, ^62^ and A‐804598.^53^ Apart from these, selective brain penetrant P2X7R antagonist JNJ‐54175446 and JNJ‐55308942 have been transitioned into clinical placebo‐controlled phase II studies to evaluate their effects in major depression disorder.^124^

However, hippocampal administration of P2R agonists injection (ATP 100 nmol/rat or BzATP 10 nmol/rat) for 3 weeks resulted in depressive‐like behavior similar to that in stress exposure.^61^ Many researchers have also explored the possible mechanisms through which P2X7R may be involved in depression, including neuroinflammation, neuroexcitability, pyroptosis, etc. P2X7R stimulation has also been reported to increase the release of glutamate and γ‐aminobutyric acid (GABA)^121^, ^125^, ^126^ while reducing the uptake of these transmitters,^127^ leading to excitatory toxicity. Elevated glutamate levels have been reported to be associated with stress and depression, with related mechanisms involving neuroinflammatory processes, reduced levels of neurotrophic factors, decreased neurogenesis, and impaired neuroplasticity.^128^, ^129^ Pereira et al. found that when exposed to stress, P2X7R may be coupled with neuronal nitric oxide synthesis in the prefrontal cortex, leading to depressive‐like behavior by promoting the release of NO in the limbic region of the brain,^130^ which is consistent with the evidence that inhibition of NO synthesis can produce anti‐depressant‐like behavior.^131^ Chai et al. found that salidroside profoundly mediated corticosterone or lipopolysaccharide‐induced depressive behavior and the improved synaptic plasticity by upregulating the expression of BDNF gene through P2X7/NF‐κB/NLRP3(NF‐κB, nuclear factor kappa‐B; NLRP3, NOD‐like receptor thermal protein domain associated protein 3) mediated pyroptosis.^132^

In conclusion, there is substantial evidence implying that P2X7R regulates the inflammatory body NLRP3/NLRP1, glutamate, GABA, NO release, and BDNF, and these mechanisms involve inflammation, neuroexcitability, pyroptosis, and more.

This part is contrary to our point that insufficient ATP release could lead to depression and that ATP supply may alleviate depression. The possible explanation is that high concentrations of eATP(EC_50_ > 100 μM)act as a danger signal sensed by P2X7R,^133^ leading to the occurrence of depression. In this context, we emphasize that the concentration of eATP may contribute to different results. It is possible that within the normal range of ATP concentration, insufficient ATP release could lead to reduced eATP concentration, ultimately resulting in the development of depression. eATP supply may alleviate depression. However, in the condition of high eATP concentration (EC50 ≥ 100 μmol ·L^− 1^), P2X7R is bound by eATP, activating the inflammatory pathway and leading to depression.

Additionally, the released eATP can be rapidly converted into adenosine by ecto‐nucleosidases in less than a minute,^134^ actually in milliseconds within synapses.^135^ Both A1 receptors and A2a receptors have been found to play an important role in depression.^63^, ^64^, ^117^, ^118^ However, our understanding of the intricate interplay between adenosine and various other neuromodulation systems remains limited, and we know even less about the interaction between ATP and adenosine signaling‐two sister worlds that seem to avoid looking into each other.^136^ Nonetheless, it is increasingly recognized that abnormal expression of eATP and regulation of ATP receptors may lead to depression^137^ and eATP could be a novel therapeutic target for depression.^138^

#### 2.4.2 ATP+P2X2R and depression

P2X2 receptors are ligand‐gated ion channels that open a cation‐selective pore in response to ATP binding to its large extracellular domain.^139^ Gao Tianming's team discovered that decreased ATP release from astrocytes led to depressive‐like behaviors in mice.^41^ Conversely, exogenous ATP administration or endogenous activation of astrocytes promoted ATP release, revealing the potential cellular and neural circuit mechanism of ATP regulation of depressive‐like behaviors. ATP deficiency via the P2X2 receptor inhibits GABAergic interneurons, resulting in reduced GABAergic inhibition of mPFC neurons projecting to LHb.^57^ Consequently, the activity of this pathway is correlated with the occurrence of depressive‐like behaviors.^57^ Additionally, their research also suggests that astrocytes‐derived ATP may regulate autism‐like disorders through the P2X2 receptor.^140^

Cao and colleagues have identified that P2X2 receptors are increased in the mPFC of susceptible mice in Chronic Social Defeat Stress (CSDS). Conditional knockout of P2X2 receptors in pyramidal neurons promoted resilience of chronic stress‐induced depressive‐like behaviors, whereas pyramidal neurons ‐ specific gain of P2X2 in the mPFC increased vulnerability to depressive‐like behaviors.^58^

Lower log‐transformed P2X2 gene expression was observed in the peripheral blood of MDD than in that of Healthy volunteers, suggesting a significant relationship between P2X2 mRNA expression and depression.^141^

It is shown that activation of postsynaptic P2XRs by exogenous ATP or noradrenaline‐dependent glial release of endogenous ATP decreases the amplitude of miniature excitatory postsynaptic currents and α‐amino‐w3‐hydroxy‐5‐methyl‐4‐isoxazolpropionate (AMPA)‐evoked currents in cultured hippocampal neurons. It has also been observed a P2X‐mediated depression of field potentials recorded in the hippocampal CA1 region from brain slices.^142^ P2X2Rs trigger dynamin‐dependent internalization of AMPA receptors(AMPARs), leading to the reduced surface AMPARs in dendrites and at synapses. The alteration of AMPAR requires calcium influx through opened ATP‐gated channels and phosphatase or calcium‐calmodulin‐dependent protein kinase II (CamKII) activities.^142^ These findings indicate that postsynaptic P2XRs play a critical role in regulating the surface expression of AMPAR, thereby regulating the synaptic strength.^142^

It is reported that both P2X2 and P2X4 interact with NMDARs in an inhibited manner.^143^ Ca^2+^ entry through P2X2Rs or P2X4Rs is sufficient to trigger internalization of AMPARs in vitro. However, ATP‐induced enduring decrease of miniature excitatory postsynaptic currents (mEPSCs) and ATP‐induced AMPAR internalization in hippocampal neurons are blocked by pyridoxalphosphate‐6‐azophenyl‐2′,4′‐disulphonic acid.^142^

It can be inferred that ATP might influence MDD through two distinct pathways involving P2X2 receptors. Firstly, ATP is believed to regulate GABA synaptic transmission, a process that has been proven to modulate autism spectrum disorder‐like behavior.^140^ Secondly, ATP plays a significant role in the function, regulation, and downstream signaling of calcium channels,^144^ contributing to depression.^145^ In conclusion, the regulation of ATP on P2X2 receptor may be a target for the treatment of mental diseases such as depression.

#### 2.4.3 ATP+P2X4R and depression

The P2X4 receptor (P2X4R) is an ATP‐gated cation channel that is highly permeable to Ca^2+^ and is widely expressed in neuronal and glial cell types throughout the central nervous system. A growing body of evidence indicates that P2X4R plays key roles in several central disorders.^146^ Among the seven P2X subunits, P2X4R stands out as the most widely distributed in various cell types throughout the body.^147^ Nevertheless, P2X4R expression in the brain is sparse^148^ and its contribution to the synaptic modulation in normal conditions remains debated.^99^, ^149^

Depression, bipolar disorder, schizophrenia, and anxiety are neuropsychiatric disorders characterized by impaired dopamine (DA) homeostasis. As previously reported, P2X4R has been implicated in the regulation of DA homeostasis and sensory–motor gating^150^ and there is increasing evidence implying that P2X4R plays a critical role in psychiatric disorders.^59^, ^60^, ^151^ Consistent with an involvement of P2X4 receptors, ivermectin (P2X4‐potentiating drug^152^) has been found to induce anxiolytic‐like and depressive‐like behavior in mice.^60^ Remarkably, data from a new conditional P2X4 internalization‐defective knock‐in mouse, namely P2X4mCherryIN, which displays an increase in the number of P2X4 receptors at the surface of targeted cells, further supports the relation between neuronal P2X4 and anxiety‐like behavior as well as in memory.^153^ The anxiolytic effects resulting from a selective increase in surface P2X4 density in excitatory forebrain neurons of mice further emphasize the role of neuronal P2X4 in anxiety.^153^ Another study, focusing on the role of P2X4 in ischemic stroke using P2X4‐KO mice, has shown that P2X4 deletion predisposes animals to chronic depression‐like behavior after stroke.^59^ Significantly in this context, a recent study revealed that the potentiation of P2X4 by ivermectin leads to DA hyperactivity and the disruption of information processing, possibly due to the potential perturbation of the interaction between P2X4 and DA receptors.^154^ Collectively, these findings suggest that P2X4 antagonists could serve as novel anti‐psychotic treatments for psychiatric disorders arising from sensorimotor gating impairments linked to the disruption of DA homeostasis.

In conclusion, these findings provide further evidence for the connection between eATP and depression and the regulation of ATP on the P2X4 receptor may be a target for the treatment of mental diseases such as depression.

### 2.5 ATP+ adenosine and depression

eATP can be converted into adenosine by ecto‐nucleotidase in milliseconds^135^ in a channeling manner.^134^ The four adenosine receptor subtypes A1, A2A, A2B, and A3 are G‐protein coupled. Typically, A1 and A3 receptors in brain couple to the Gi/o family of G‐protein, which inhibit the synthesis of cyclic AMP.^155^ The concentration of extracellular adenosine in the brain is determined by the hydrolysis of ATP released from neurons or astrocytes and by transport through equilibrative nucleoside transporters.^156^ Under neuropathological conditions such as ischemia, trauma, excitotoxicity, neurodegeneration, neuroinflammation, and epilepsy, the extracellular concentration of adenosine in the brain can rise rapidly from nanomolar to micromolar levels, which can have both beneficial and detrimental effects on the progression of the illness.^157^ Among these receptors, A1 receptors are most abundant and homogenously distributed in the brain, providing an inhibitory and general neuroprotective “tone.”^158^ Neuronal and glial adenosine A2A receptors play a critical role in neuropsychiatric disturbances.^157^ Both A1 receptors and A2A receptors have been found to play an important role in depression.^63^, ^64^, ^117^, ^118^

Both A1R and A2AR modulate hippocampal glutamate release and NMDA‐dependent LTP but aging affects the density of both A1R and A2AR.^105^ It is observed that adenosine modulates hippocampal LTP through activation of adenosine A1R mostly only in young animals than in adult animals and A2 receptors in synaptic plasticity are mostly observed in aged rather than young animals.^105^ What's more, A1 receptors in synaptic plasticity are under tight repression by A2A receptor engagement, and A2AR could play a key role in dampening A1R during high‐frequency induction of hippocampal LTP.^159^

The beneficial effects of 12‐hour sleep deprivation in experimentally induced depressive‐like behavior of mice require astrocytic signaling to A1 receptors, which may provide a novel pathway for the development of anti‐depressants.^118^

Adenosine antagonists such as caffeine (the non‐selective adenosine antagonist) and SCH412348 (the selective adenosine antagonist), have been shown to be effective in reversing signs of behavioral despair in the tail suspension and forced swim tests, two screening procedures predictive of anti‐depressant activity.^63^, ^64^, ^65^ The impact of a non‐toxic concentration of caffeine on synaptic transmission and plasticity in excitatory synapses of the mouse hippocampus is critically dependent on the presence of endogenous extracellular adenosine.^160^ The antagonism of adenosine receptors is indeed the main mechanism operated by non‐toxic concentrations of caffeine to modify information processing in neuronal networks while directly excluding a role for GABA or ryanodine receptors as targets of caffeine at non‐toxic concentrations.^160^ Under toxic concentrations of caffeine, it is possible that other mechanisms apart from the adenosine modulation system, namely ryanodine receptor‐induced calcium release, may be engaged by caffeine to modify synaptic transmission and plasticity.^161^, ^162^, ^163^

It is worth noting that epidemiological studies have revealed a relationship between caffeine consumption and reduced risk of depression^164^ and some researches have demonstrated the use of caffeine as a self‐medication among depressed patients.^165^ However, in human data, the therapeutic effects of caffeine depend upon the administered dose, as high doses of caffeine and theophylline not only do not improve depressive symptoms, but can in fact promote anxiety.^166^, ^167^ Therefore, it is advisable for individuals with depression and anxiety syndromes to take caffeine in appropriate doses.

eATP can be converted into adenosine quickly. We think that it is hard to distinguish between ATP and adenosine pathways. Notably, brain noxious stimuli trigger a sustained increase of eATP, which plays a key role as a danger signal in the brain. The concept of ATP as a danger signal implies not only the release of ATP but also the involvement of purinergic receptors in brain disorders, which has mostly been documented for P2X7R, P2Y1R, and A2AR.^168^ Metabolic stress, characteristics of brain dysfunction, is associated with increased extracellular levels of both ATP and adenosine, both of which act as danger signals in the brain with potential deleterious effects. It is unlikely that any experimental setting can isolate the contribution of ATP from that of adenosine and the different roles fulfilled by ATP and adenosine.^136^ Therefore, we think that it is not rigorous and incomplete to discuss the role of eATP in depression in isolation from the contribution of adenosine and P2X receptors.

Additionally, high levels of adenosine reflect high energy expenditure, leading to the reduced levels of eATP. The adenosine A2A agonist CGS21680 has been reported to decrease the affinity of dopamine D2 receptors for dopamine.^169^ In the context of mood depression, A2A receptors could also control the release of glutamate^170^ and NMDA^106^ receptor function. In contrast to the particular interaction in the basal ganglion, A2A receptors could modulate the dopamine D2 receptor‐mediated inhibition of synaptic transmission in the mouse prefrontal cortex.^171^

We have limited knowledge about the intricate interplay of adenosine with other various neuromodulation systems, and even less about the interplay between ATP and adenosine signaling, two sister worlds that seem to avoid looking into each other.^136^

In summary, we think that the elevated levels of adenosine in brain tissue can lead to depression, irritability and fatigue, and replenish energy can convert adenosine to adenosine triphosphate (ATP), finally alleviating depressive symptoms.

## 3 TREATMENT PROSPECTS

Given that currently available anti‐depressants primarily impact monoaminergic signaling, specifically by inhibiting the reuptake of serotonin (5‐hydroxytryptamine) and norepinephrine (NA), exploring alternative molecular targets other than monoamines may help identify biomarkers for depression. This could lead to the development of drugs with better therapeutic efficacy. Currently, some of the potential pharmacological treatments for depression include mood stabilizers,^172^ A2A receptor antagonists,^173^, ^174^ ATP‐sensitive P2X7 receptor antagonists,^54^, ^62^ P2X2 receptor agonists,^57^ ATP‐sensitive potassium channel opener etakalin,^175^ and nutrients^176^ (e.g., omega‐3 fatty acids, antioxidants like vitamin C and zinc, members of the vitamin B family(vitamin B12 and folic acid) and magnesium), therapies targeting NLRP3 inflammasomes, interferon therapies,^177^ tumor necrosis factor‐α receptors, statins, interleukin‐β antagonists, acetyl L‐carnitine therapy,^178^ ketamine^18^, ^67^, ^179^, ^180^, ^181^ and so on. Many studies have suggested that eATP can play an anti‐depressant role, and ATP supplement can relieve depression, suggesting that eATP therapy could be beneficial for depression. Research has also indicated that P2X2 receptor may play an anti‐depressant role and P2X2 receptor agonist is expected to treat depression.^57^ eATP treatment or enhancement of Calhm2 function^182^ is also a possible therapeutic avenue for depression therapy. CD39 is an enzyme that is responsible, together with CD73, for a cascade converting ATP into ADP and cyclic AMP.^183^ Hippocampal CD39 has been involved in depressive‐like behavior induced by CSDS through hydrolyzing eATP. This suggests that CD39 could be a promising new target for the treatment of depression, which can be realized by CD39 gene silencing/pharmacological inhibition or ATP supplementation.^48^ Moreover, increased synaptic ATP release, coupled to CD73‐mediated formation of extracellular adenosine, could contribute to mood and memory dysfunction triggered by repeated restraint stress.^49^ Therefore, interventions aimed at decreasing ATP release and CD73 activity could be novel strategies to mitigate the burden of repeated stress.^49^ Other studies have demonstrated that the downregulation of Panx1 in CSDS models results in the deficiency of eATP in mPFC, leading to depressive‐like behaviors. This suggests that Panx1 may be a key target for anti‐depressant therapy, particularly in the context of mefloquine‐induced depression.^50^ Furthermore, some anti‐depressant drugs such as SSRIs, have been discovered to inhibit the activation of microglia and promote the release of ATP, BDNF, and vascular endothelial growth factor from astrocytes to restore neurogenesis.^47^

## 4 DISCUSSION

Several pieces of evidence have indicated that decreased ATP release or increased ATP catabolism may be important factors involved in the development of depression. On the other hand, elevated eATP levels can exert an anti‐depressant effect, eATP supply can exert an anti‐depressant effect, providing a new strategy for the treatment of depression. However, while reviewing numerous studies, we encountered some contradictory findings. For instance, the activation of the P2X7 receptor has been confirmed to be closely linked to depression, and its activation typically requires a high concentration of ATP (EC50 ≥ 100 μmol L^−1^), often due to exposure to stressful conditions. In contrast, many other studies suggest that insufficient ATP release from astrocytes may lead to depression, leaving us puzzled about ATP's actual role in depression. First, it is important to recognize that stress is a complex process, and the relationship between stress and depression is not clear. Acute exposure to stressors could result in depression.^42^, ^46^ When exposed to acute stressors, microglial cells can be activated, leading to ATP release (combined with P2X receptors) and the activation of the inflammatory response. The inflammatory response is known to be associated closely with depression. Conversely, chronic stress is often accompanied by mitochondrial structural damage,^58^, ^184^, ^185^ potentially leading to the reduced ATP production and depression. Currently, chronic stress models are predominantly used to study depression, and the existence of mitochondrial structural damage has been confirmed, along with an increasing body of research demonstrating the role of reduced ATP release from astrocytes in depression. Secondly, some articles have shown that ATP release from astrocyte could play an important role in the anti‐depressant effects. In microglia, the interaction between ATP and purinergic receptors may facilitate the activation of downstream inflammatory responses, potentially leading to depressive‐like behaviors. We speculate that these discrepancies may be attributed to variations in eATP concentration. Considering the mounting evidence supporting the role of ATP reduction in depression, it is reasonable to believe that the modulation of eATP levels or the inhibition of its degradation may emerge as a novel depression treatment strategy in the future. This approach could be particularly beneficial for patients who do not respond to monoamine anti‐depressants.

To conclude, a growing body of researches lends the credibility to the notion that eATP plays an important role in depression. The development of drugs that increase eATP levels may be a promising strategy for treating depression in the future. However, several challenges need to be addressed:

(1) Although there is increasing evidence implying that the dysregulation of eATP is related to the pathophysiology of depression, the cellular and neural circuitry mechanisms underlying how ATP regulates depressive‐like behavior require further investigation. Therefore, a large number of studies are needed to elucidate the role of eATP in depression.

(2) ATP has demonstrated a short duration of anti‐depressant effects in mice with a high efficiency,^41^ but it remains to be seen whether this effect could translate to human. To establish the precise correlation between eATP and depression and the feasibility of using eATP to treat depression in humans, numerous researches involving human brain samples are warranted.

(3) More targeted anti‐depression drugs, such as P2X2 receptor agonists, need to be developed.

In summary, many studies have shown that abnormal ATP release especially from astrocytes is associated with depression. In the future, more research about depression is needed to explore the relationship of eATP released not only from astrocytes but also from neurons, synaptic, and so on. What's more, considering the interactional complexity of ATP and purinergic signaling pathways, we need to specify the role of eATP rather than adenosine and P2X receptors in depression(although these two sisters [ATP and adenosine] worlds that seem to avoid looking into each other), aiming to increase the persuasive effects of eATP in depression. Most of the researches are confined to animal studies at present. Therefore, what we need to do is to conduct a large number of studies to clarify the mechanism of the relationship between eATP and depression, carry out more clinical studies on the treatment of depression patients with ATP, and develop targeted drugs to guide the treatment of depression.

## 5 CONCLUSION

Depression is a severe mental disorder whose underlying mechanisms remain inadequately understood. Considering that a substantial portion of individuals with depression do not respond to traditional monoamine‐based anti‐depressant therapies, there is an urgent imperative to delve deeper into the intricacies of depression mechanisms. Several studies have demonstrated that eATP may play a crucial role in depression, especially from the angle of ATP release from astrocytes. We present the point that the reduction in ATP release could serve as a pivotal role in the pathogenesis of depression. However, there are few researches on the mechanism of eATP and depression, and most of them are confined to animal studies at present. Nonetheless, the research on the interplay between eATP and depression remains limited, predominantly confined to animal studies at present. Therefore, further research is needed to elucidate the mechanisms underlying the relationship between eATP and depression. This would entail conducting a substantial number of clinical studies involving ATP treatment for individuals with depression, ultimately paving the way for the development of targeted medications designed to guide the treatment of depression.

## AUTHOR CONTRIBUTIONS

Kaixin Wang and Shiqian Huang contributed substantially to the article concept and manuscript writing. Kaixin Wang, Shiqian Huang, Daan Fu, Xinxin Yang, Lulin Ma, Tianhao Zhang, Wenjing Zhao, Daling Deng, Yuanyuan Ding, Yanyan Zhang, and Li Huang retrieved literature and reviewed themanuscript. Xiangdong Chen revised and approved the final version before submission. All the listed authors have participated actively in the study, and have read and approved the final manuscript.

## CONFLICT OF INTEREST STATEMENT

Authors initials (e.g., WKX, HSQ, FDA, YXX, MLL, ZTH, ZWJ, DDL, DYY, ZYY, HL, CXD)—no competing interests declared.

## Data Availability

Data sharing not applicable to this article as no datasets were generated or analysed during the current study.
